# Obstetric and Gynaecological Challenges and Outcomes in Women and Girls With Glanzmann's Thrombasthenia

**DOI:** 10.1111/hae.70030

**Published:** 2025-03-23

**Authors:** Deborah Obeng‐Tuudah, Ahmad Tarawah, Melike Ozkan, Rezan Abdul‐Kadir

**Affiliations:** ^1^ Department of Obstetrics and Gynaecology Katharine Dormandy Haemophilia and Thrombosis Centre, The Royal Free NHS Hospital and Institute for Women's Health University College London London UK; ^2^ Madinah Hereditary Blood Disorders Center King Salman Medical City Madinah Saudi Arabia

**Keywords:** Glanzmann's thrombasthenia, heavy menstrual bleeding, platelet transfusion, postpartum haemorrhage, recombinant factor VIIa, women and girls

## Abstract

**Introduction:**

Glanzmann's thrombasthenia (GT) is an inherited platelet function disorder that may manifest with significant bleeding symptoms; in women and girls (W&Gs), heavy menstrual bleeding (HMB) is very common. GT in pregnancy is associated with an increased risk of postpartum haemorrhage (PPH).

**Aim:**

This study highlights the gynaecological and obstetric challenges experienced by W&Gs with GT, and reviews available treatment options.

**Methods:**

Data regarding 38 W&Gs with GT were analysed from the ISTH REDCap registry, an international multi‐centre database.

**Results:**

Among 38 W&Gs, 76% of Middle Eastern ethnicity, 100% reported HMB; 92% HMB since menarche, and 82% presented with acute HMB and were treated with platelets and packed red blood cells (pRBCs) transfusions in addition to hormonal therapies. Management of chronic HMB required a combination therapy including antifibrinolytics (tranexamic acid [TXA]), hormonal therapies, and recombinant factor VIIa (rFVIIa); rFVIIa was used in 50% of W&Gs. In 16 pregnancies, PPH was reported in 63% of deliveries, of which 83% required blood and platelet transfusions. Despite prophylactic haemostatic agents during labour and delivery in 8/9 pregnancies of women with known GT diagnosis, 78% experienced PPH. Thirty‐one percent of neonates developed neonatal alloimmune thrombocytopenia (NAIT).

**Conclusion:**

HMB and PPH are common bleeding complications in GT. Effective management of HMB and PPH in W&Gs with GT is challenging but can be achieved by a multidisciplinary team, often requiring a combination of haemostatic agents with hormonal therapies. Use of rFVIIa may limit the need for platelet transfusion, thus reducing alloimmunisation and the risk of developing NAIT.

## 1 Introduction

Glanzmann's thrombasthenia (GT) is an autosomal recessive inherited platelet function disorder (IPFD) that is caused by decreased, absent or defective glycoprotein (GP) IIb/IIIa expression [1]. GP IIb/IIIa is a receptor found on platelet membranes. It binds fibrinogen to mediate aggregation of activated platelets, an important process in haemostasis leading to cessation of bleeding after vascular injury/trauma or surgery [2].

GT is rare, with a global incidence of 1/1,000,000 population [3], and distributed worldwide with predominance in populations where consanguinity is common (e.g., prevalence of 1/10,000 in Madinah, Kingdom of Saudi Arabia) [4, 5]. Although heterozygous carriers of the GP IIb/IIIa mutations are asymptomatic, homozygous or compound heterozygous patients have significant bleeding symptoms. Epistaxis, easy bruising and purpura, gingival bleeding and heavy menstrual bleeding (HMB), previously referred to as menorrhagia, are the most common symptoms in patients [6]. HMB has been defined as an estimated blood loss of > 80 mL/cycle. Clinically, HMB is defined as excessive uterine bleeding, which negatively impacts the physical, emotional, social and material quality of life of women and girls (W&Gs) [7, 8].

Evaluating bleeding risk in patients with GT involves careful clinical assessment, including bleeding history, family history and/or parental consanguinity [9]. The International Society on Thrombosis and Haemostasis bleeding assessment tool (ISTH BAT), is a standardised questionnaire used to assess the severity of bleeding symptoms and response to treatment in inherited bleeding disorders [10].

Management of GT may be challenging due to disease rarity with recommendations and guidelines developed from expert opinions, case reports and registry data [9, 11–15]. Treatment selection for GT may vary according to bleeding severity, treatment availability and responsiveness. Mild to moderate bleeds may be managed with local/adjunctive measures like compression, topical thrombin and/or antifibrinolytics (e.g., tranexamic acid [TXA]), whilst severe bleeds may require additional systemic haemostatic agents, including platelet transfusion or recombinant factor VIIa (rFVIIa) (a bypassing agent), in combination or alone [14, 16]. The gold standard treatment for GT is platelet transfusion. However, this treatment is associated with risks of alloimmunisation, which occurs in up to 30% of patients with GT, leading to ineffective future transfusions. Allergic reactions are also possible [16, 17, 18].

For management of HMB in W&Gs with GT, important considerations include cultural and social perspectives regarding the menstrual cycle, particularly in Middle Eastern countries, where treatments that halt the menstrual periods may be unacceptable [9]. This has led to the development of a special Madinah protocol, which includes the use of a combination treatment of TXA, combined oral contraceptive pill (COCP) and rFVIIa [9].

Although there are limited data on GT in pregnancy and delivery, associations with severe postpartum haemorrhage (PPH) have been reported [19, 20]. This necessitates optimum risk assessments and a comprehensive management plan for pregnancy, delivery, and the postpartum period by a multidisciplinary team (MDT) (e.g., haematologists, obstetricians, anaesthetists and neonatologists) [9, 20].

We present data collected from the ISTH REDCap registry, focusing on the presentation, management and outcomes of obstetric and gynaecological issues in W&Gs with GT [21].

## 2 Methods

### 2.1 Patients and Study Design

The ISTH REDCap registry is an international multi‐centre registry, designed to capture anonymised data on clinical presentation, management and outcomes of W&Gs with IPFD [21]. Data on all W&Gs with a confirmed diagnosis of GT (*N* = 38) were extracted from the registry from 12 March 2018 to 30 September 2022. This retrospective study was authorised by The Royal Free NHS Hospital (United Kingdom), and the Madinah Hereditary Blood Disorders Center (Kingdom of Saudi Arabia). Data collected included demographics and assessment of bleeding using the ISTH BAT bleeding score, and menstrual history. Endpoints include frequency of HMB and PPH, pregnancy complications (miscarriage and antepartum haemorrhage [APH]), treatment options and their efficacy in the management of HMB and PPH, high dependency unit (HDU) admission, and neonatal complications.

## 3 Results

### 3.1 Patients

A total of 38 W&Gs were included in the analysis. Patient baseline characteristics (including bleeding outcomes) and demographics are presented in Table 1. Not all aspects of collected data were available for all W&Gs in the registry. Seventy‐six percent (29/38) of W&Gs were of Middle Eastern ethnicity, 89% (34/38) had a positive family history for GT, and parental consanguinity was present in 87% (33/38). The median age at the time of data collection for the registry was 21 years (range, 13–71).

**TABLE 1:** Demographic and Gynaecological characteristics in women and girls with Glanzmann's thrombasthenia.

Characteristic, units	Values
Number of patients, *N*	38
**Age**	
Age at diagnosis, median (range), years	7 (0–26)
Age at data collection, median (range), years	21 (13–71)
**Ethnicity and family history**	
Middle Eastern^a^, *n* (%)	29 (76)
South Asian^b^, *n* (%)	3 (7.9)
Australian, *n* (%)	1 (2.6)
Unknown, *n* (%)	5 (13.2)
Positive family history of GT, *n*/*N* (%)	34/38 (89.5)
Parental consanguinity, *n*/*N* (%)	33/38 (86.8)
**Baseline bleeding outcomes**	
ISTH bleeding score, median (range), years	8 (4–22)
Age at menarche, median (range), years	12 (9–19)
Length of menstrual bleeding, mean (range), days	9 (5–21)
HMB reported, *n*/*N* (%)	38/38 (100)
HMB since menarche, *n*/*N* (%)	35/38 (92.1)
**Consultation**	
Consultation for HMB, *n*	36^c^
Gynaecologist, *n* (%)	27 (75)
GP/family doctor, *n* (%)	1 (2.8)
No consultation for HMB, *n* (%)	8 (22.2)
Patients with no regular treatment for HMB, *n*/*N* (%) Presented with acute menstrual bleeding, *n*/*n* (%)	10/38 (26.3) 8/10 (80)
Attendance at emergency department for acute episodes of HMB, *n*/*N* (%)	31/38 (81.6)
**Anaemia in patients with HMB**:	
Anaemia, *n*/*n* (%)	24/35 (68.6)
Treatment:	24
Oral iron, *n*/*n* (%)	24/24 (100)
Intravenous iron, *n*/*n* (%)	3/24 (12.5)
pRBCs, *n*/*n* (%)	2/24 (8.3)
**Ovarian bleeding**	3/38 (7.9)
Patient 1 treatment:	
Recurrent ovarian haemorrhagic cyst	TXA
Haemoperitoneum^d^	TXA + norethisterone + desmopressin + platelets
Patient 2 treatment:	
Recurrent ovarian haemorrhagic cyst	TXA + 6 months Buserelin nasal spray
Patient 3 treatment:	
Acute haemoperitoneum^d^	pRBCs + platelets + laparotomy to stop source of bleeding and clean the abdomen

Abbreviations: GP, general practitioner; GT, Glanzmann's thrombasthenia; HMB, heavy menstrual bleeding; ISTH, International Society on Thrombosis and Haemostasis; *n*, number of patients; *N*, total number; pRBCs, packed red blood cells; total number of patients; rFVIIa, recombinant factor VIIa; TXA, tranexamic acid.

^a^ Countries in the Middle East include Saudi Arabia, Iraq, Iran, Turkey and Israel.

^b^ Countries in South Asia include India, Pakistan Bangladesh and Sri Lanka.

^c^ No information available in 2 W + G.

^d^ Associated with ovulation.

**FIGURE 1:**
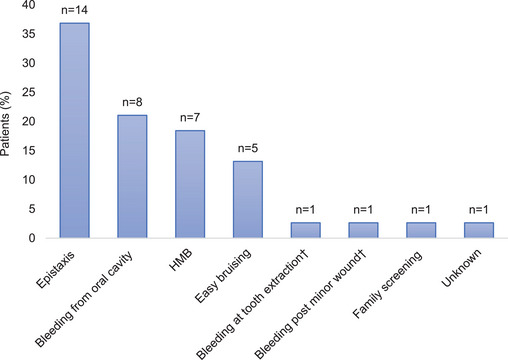
Main presenting symptoms leading to diagnosis of Glanzmann's thrombasthenia in women and girls (*N* = 38). Easy bruising is defined as cutaneous bruises, including ecchymosis. HMB, heavy menstrual bleeding; *N*, total number of patients; *n*, number of patients; ^†^excessive/prolonged bleeding.

**FIGURE 2:**
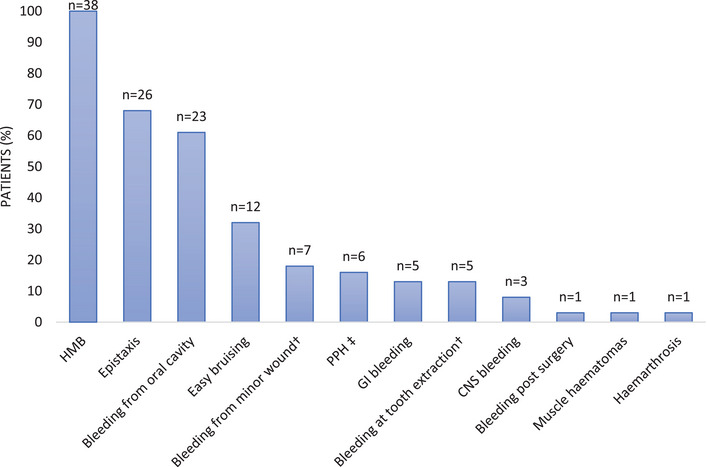
Frequently reported bleeding symptoms of women and girls with Glanzmann's thrombasthenia (*N* = 38). The percentage illustrated represents 6/38, using the denominator (*N* = 38) for the whole patient population for consistency. Note that most of the W&Gs have not had a pregnancy. ^†^Excessive/prolonged bleeding. CNS, central nervous system; GI, gastrointestinal; HMB, heavy menstrual bleeding; *N*, total number of patients; *n*, number of patients; PPH, postpartum haemorrhage; ^‡^history of PPH was reported in 6 out of 7 women (86%) with viable pregnancies. Easy bruising is defined as cutaneous bruises, including ecchymosis.

The median age at diagnosis was 7 years (0–26); 68% (26/38) of W&Gs were diagnosed before menarche. The main presenting symptoms leading to diagnosis were mucocutaneous bleeding, with epistaxis (14/38, 37%) and bleeding from the oral cavity (8/38, 21%) (Figure 1). Despite positive family history in 34 W&Gs, family screening led to diagnosis only in one case. HMB led to diagnosis in 7/38 (18%) and was the most common bleeding symptom, reported in 38/38 (100%) W&Gs, followed by epistaxis (26/38, 68%), bleeding from the oral cavity (23/38, 61%), easy bruising (12/38, 32%) and other symptoms reported in Figure 2. The median ISTH BAT score was 8 (4–22).

Anaemia was common in this population at 24/35 (69%); all W&Gs required long‐term iron treatment (oral [24/24, 100%] or intravenous [3/24, 12.5%]). Eight percent (2/24) of W&Gs required packed red blood cell (pRBC) transfusion (Table 1).

### 3.2 Gynaecology Findings

The median age of menarche was 12 years (9–19), and the median length of menstrual bleeding was 9 days (5–21). HMB from menarche was reported in 35/38 (92%) W&Gs, with all reporting HMB at the end. At least one presentation to the emergency department with acute HMB was reported for 31/38 (82%) W&Gs. Of the 10 W&Gs (26%) without regular treatment for HMB, 80% presented with acute episodes of HMB. A summary of gynaecology findings is presented in Table 1.

#### 3.2.1 Management of HMB

W&Gs presenting with acute HMB (31/38, 82%) received a combination therapy encompassing hormonal therapy with progestogens (30/31, 97%) and COCP (9/31, 29%), platelets (18/31, 58%), antifibrinolytics (e.g., TXA) (17/31, 55%) and rFVIIa (4/31, 13%). Surgery (hysteroscopy with dilatation and curettage) was used in 1/31 (3%). Blood transfusion was required in 16/31 (52%), and iron treatment in 17/31 (55%). The median total numbers of units of platelets and pRBCs used to manage acute HMB in these W&Gs were 8 (1–29) and 3 (1–19), respectively. See Table S1.

Effective treatment for chronic HMB (as reported by patient satisfaction during follow‐up) was reported by 26 W&Gs (Table 2). Single agent therapy with COCP or norethisterone was effective in 5/26 (19%), and a two‐agent combination therapy (TXA and COCP) in an additional 3/26 (12%). The remaining 18/26 (69%) needed a three‐ or four‐agent combination treatment, whereby TXA with COCP and rFVIIa were used in 13/26 (50%), and TXA with COCP and norethisterone in 2/26 (8%) W&Gs. In W&Gs where control of HMB had proved difficult with the above triple therapy, TXA with short‐term therapy of gonadotropin‐releasing hormone (GnRH) for 6 months to suppress ovarian function and induce amenorrhoea with add‐back hormonal therapy with tibolone 1/26 (4%) plus COCP and Norethisterone 2/26 (8%) was found to be effective.

**TABLE 2:** Effective treatment for chronic HMB in women and girls with Glanzmann's thrombasthenia (*n* = 26).

Treatment agents	Number of patients, *n*/*n* (%)
Single agent treatment,	
COCP or norethisterone	5/26 (19.2)
Combination of two treatments,	
TXA + COCP	3/26 (11.5)
Combination of three treatments, *n*	
TXA + COCP + norethisterone	2/26 (7.7)
TXA + tibolone + GnRH (6 months)	1/26 (3.8)
TXA + COCP + rFVIIa (Madinah protocol)	13/26 (50)
Combination of four treatments, *n*	
TXA + COCP + norethisterone+ short term GnRH (with add‐back tibolone)	2/26 (7.7)

Abbreviations: COCP; combined oral contraceptive pill; GnRH; gonadotropin‐releasing hormone; HMB, heavy menstrual bleeding; *n*, number of patients; rFVIIa, recombinant factor VIIa; TXA, tranexamic acid.

### 3.3 Obstetric Findings

Nineteen pregnancies were documented in seven women, potentially attributable to the young median age (21 years) of this cohort. Information on obstetric outcomes is presented in Table 3. Three miscarriages were reported in three women, with a known diagnosis of GT at that time in 2/3. All three miscarriages led to heavy bleeding despite prophylaxis with combinations of TXA, platelets and rFVIIa in the women with an existing diagnosis. Bleeding during miscarriage was controlled with platelet transfusion and rFVIIa in one patient, and with platelet transfusion, TXA, rFVIIa and pRBC transfusion in the other.

**TABLE 3:** Obstetric outcomes in women with Glanzmann's thrombasthenia.

Characteristic, units	Values
Total pregnancies in 7 women, *n*	19
Termination of pregnancy^a^, *n*	0
Miscarriage rate, *n*/*n* (%)	3/19 (15.8)
Viable ongoing pregnancy, *n*	16
Bleeding in early pregnancy rate^b^, *n*/*n* (%)	1/16 (6.3)
Bleeding in late pregnancy (APH)^b^	1/16 (6.3)
Presence of pre‐eclampsia, *n*	0
Presence of SGA, *n*	0
Positive antiplatelet antibodies^c^, *n*	3
**Gestational age at delivery in weeks, mean**	38.9
Term, *n*/*N* (%)	≥ 37 weeks 14/16 (87.5%)
Preterm, *n*/*N* (%)	< 37 weeks 2/16 (12.5%)
**Diagnosis of GT at miscarriage, *n* (%)**	
Known	2 (66.7)
Unknown	1 (33.3)
Regional anaesthesia, *n*	0
Spinal haematoma, *n*	0
**Mode of delivery, *n* (%)**	
Normal vaginal delivery	11 (68.8)
Ventouse/Forceps delivery	1 (6.3)
Emergency caesarean section	1 (6.3)
Elective caesarean section	3 (18.8)

Abbreviations: APH, antepartum haemorrhage; CS, caesarean section; GT, Glanzmann's thrombasthenia; IVIG, intravenous immunoglobulin; *n*, number of pregnancies; *N*, total number of pregnancies; *n*, number; SGA, small for gestational age; tranexamic acid.

^a^ There were no reports of terminated pregnancies.

^b^ The patient with APH was treated with TXA and platelets, as well as packed red blood cells when severely anaemic. She delivered at term (≥ 37 weeks) by elective CS.

^c^ Patient developed anti‐platelet antibodies in three pregnancies that were treated with IVIG.

**FIGURE 3:**
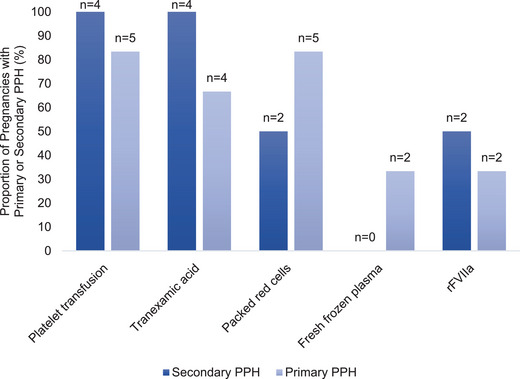
Management of primary PPH (*n* = 6) and secondary PPH (*n* = 4) in women with Glanzmann's thrombasthenia. *n*, number of pregnancies; PPH, postpartum haemorrhage; rFVIIa, recombinant factor VIIa.

**FIGURE 4:**
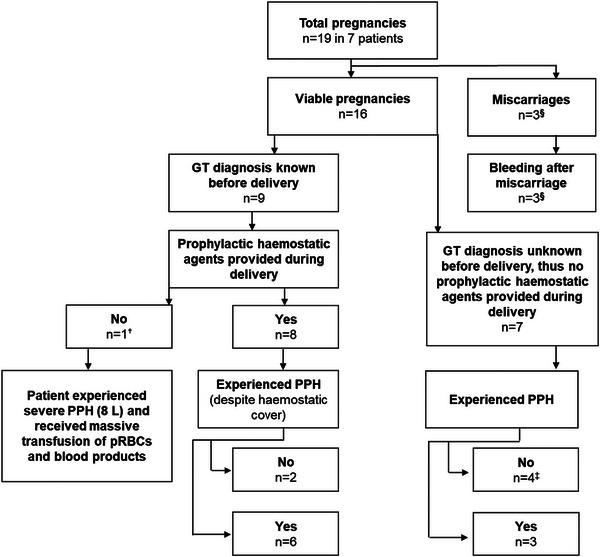
Diagnosis of Glanzmann's thrombasthenia and PPH outcome in women. GT, Glanzmann's thrombasthenia; ISTH BAT, International Society on Thrombosis and Haemostasis bleeding assessment tool; n, number of pregnancies; PPH, postpartum haemorrhage; pRBCs, packed red blood cells; ^†^prophylactic haemostatic agents were not provided during delivery despite the GT diagnosis due to an underprepared medical team; ^‡^patients had a low bleeding phenotype as noted by their lower ISTH BAT score compared to those who experienced PPH (see Table 5). This also explains why their GT diagnosis was unknown at delivery and missed in childhood; ^§^GT diagnosis was known in two cases, which were given prophylaxis before miscarriages occurred. However, heavy bleeding was experienced during all three miscarriages.

In the 16 (84%) pregnancies reaching viability stage, the GT diagnosis was unknown in seven pregnancies among three women prior to the pregnancy, four of which were contributed by one woman (Table 4 and 5). Regardless of GT diagnosis status, PPH was present after 10/16 (63%) deliveries (primary PPH, 6/10 [60%]; secondary PPH, 4/10 [40%]) (Figure 3). PPH occurred following 7/9 (78%) pregnancies with a known diagnosis of GT and following 3/7 (43%) pregnancies with an unknown diagnosis during pregnancy. Figure 4 presents an overview of PPH outcomes. More information on other obstetric outcomes, including cases of APH and development of anti‐platelet antibodies, is presented in Table 3.

Fourteen (88%) babies were born at term (≥ 37 weeks), whilst two babies were born at 34 and 35 weeks, respectively, with the majority being delivered via normal vaginal delivery (NVD) (11/16, 69%). Reasons for CS (caesarean section) (4/16) included an emergency CS due to foetal distress (1/4), a precautionary measure due to recurrent APH during pregnancy (1/4), and maternal requests (2/4). Regional anaesthesia was avoided during all deliveries. Notably, there were no cases of pre‐eclampsia or babies small for gestational age (SGA) in this group.

#### 3.3.1 Management of PPH

GT diagnosis was known in 9/16 pregnancies (56%) before delivery, of which eight received haemostatic agents during labour and delivery. One woman received no haemostatic cover due to poor antenatal planning before she presented in labour; she had a massive haemorrhage (8 L) and received multiple transfusions of platelets and blood products (Table 4, Figure 4). In the eight pregnancies during which women received prophylactic cover for PPH, 75% (6/8) of women still experienced PPH (Table 5, Figure 4), requiring further treatment.

**TABLE 4:** Haemostatic cover used during delivery and postnatal periods in pregnancies with known diagnosis of Glanzmann's thrombasthenia (*n* = 9).

Haemostatic cover	During delivery	During postnatal period
TXA, *n* (%)	0	3 (33.3)
TXA + platelets, *n* (%)	3 (33.3)	2 (22.2)
TXA + platelets + rFVIIa, *n* (%)	5 (55.5)	4 (44.4)
No cover, *n* (%)	1^a^ (11.1)	0

*Note*: GT diagnosis was unknown in the remaining seven pregnancies out of a total of 16 pregnancies.

Abbreviations: ICU, intensive care unit; *n*, number of pregnancies; rFVIIa, recombinant factor VIIa; TXA, tranexamic acid.

^a^ The patient with no cover due to poor antenatal preparation had major obstetric haemorrhage of over 8 L during delivery. She collapsed and needed a massive transfusion of packed red blood cells, platelets and clotting factors, including fresh frozen plasma, and was taken to ICU postnatally.

**TABLE 5:** Postpartum haemorrhage and haemostatic cover used for labour and delivery in W&Gs with Glanzmann's thrombasthenia (*N* = 16).

Haemostatic Cover	Total number of pregnancies	Average ISTH BAT score	PPH	No PPH
TXA + platelets	3^a^	12.6	1/3 (33.3)	2/3 (66.7)
TXA + platelets + rFVIIa	5^a^	13.6	5/5 (100%)	0
Diagnosis known, no cover	1	22	1 (100%)	0
Diagnosis unknown, *n*/*N* (%)	7^b^	6.4	3/7 (42.9)	4/7 (57.1)
Total, *n*/*N* (%)	16		10/16 (62.5)	6/16 (37.5)

Abbreviations: GT, Glanzmann's thrombasthenia; ISTH BAT, International Society on Thrombosis and Haemostasis bleeding assessment tool; n, number of pregnancies; *N*, total number of pregnancies; *n*, number of pregnancies; PPH, postpartum haemorrhage; TXA, tranexamic acid.

^a^ Eight of the nine patients that were diagnosed with GT received haemostatic cover during delivery (please see Table 4).

^b^ Patients had a low bleeding phenotype as noted by their lower ISTH BAT score compared to those with a known diagnosis.

PPH management involved combination therapy including platelets (5/6, 83%), pRBCs (5/6, 83%), TXA (4/6, 67%) and rFVIIa (2/6, 33%) to treat primary PPH. All four women with secondary PPH received platelets and TXA, plus additional rFVIIa was used in 50% (2/4) of secondary PPH cases (Figure 3). Of the same 10 women with PPH, 60% required admission to the HDU: 83% (5/6) with primary PPH and 25% (1/4) with secondary PPH. Ongoing postnatal haemostatic cover was mostly achieved with TXA, platelets, and rFVIIa (4/9, 44%) (Table 4). Hospitalisation averaged 12.5 days and lasted up to 30 days in women with primary PPH, whilst women with secondary PPH stayed for an average of 2 days.

### 3.4 Outcomes in Neonates

Of 16 neonates (8 females and 8 males), 5/16 (31%) were diagnosed with neonatal alloimmune thrombocytopenia (NAIT) at birth, three of whom were admitted to the neonatal intensive care unit due to bleeding complications, including subdural haematoma, intracranial haemorrhage, or umbilical stump oozing, with a median stay duration of 9 days (up to 16 days). The neonate with intracranial haemorrhage was treated with platelets, pRBCs and intravenous immunoglobulin. Four of 16 (25%) neonates were later diagnosed with GT, with only one being treated with platelets. Further information on neonatal outcomes is available in Table S2.

## 4 Discussion

The current study provides a comprehensive overview of real‐world data describing the outcomes experienced by W&Gs with GT, including high rates of HMB (100%) and PPH (63%) and their treatment options. Access to an MDT and careful planning in specialised centres play a role in improved patient care.

Acute HMB is very common among W&Gs with GT, with negative consequences including hospitalisation, anaemia, and the need for blood and blood product transfusion. Painful menstrual bleeding, fatigue, and the negative psychological impact associated with HMB have been reported [22]. Menstrual education, regular assessment and control of HMB, is essential to prevent such episodes and improve quality of life [22, 23].

Effective management of chronic HMB is highlighted in this study. The combination treatment of TXA, rFVIIa, and hormonal therapy is in line with guideline recommendations for HMB treatment in women with inherited bleeding disorders [23, 24]. rFVIIa is approved for bleeding symptoms in GT in select countries [16, 17]. Data from the Glanzmann's Thrombasthenia Registry showed that rFVIIa has been effectively used for GT management, particularly when initiated early and used as part of a combination haemostatic treatment [11, 12]. In addition, rFVIIa was associated with low rates of adverse events regardless of platelet antibodies and/or platelet refractoriness [11–13, 16]. TXA and various hormonal treatment options, including extended use of combined hormonal contraceptives, progestogens and/or GnRH with add‐back therapy, are also highly effective [25, 26, 27].

However, amenorrhoea and irregular bleedings associated with some hormonal treatment options may be culturally unacceptable to some women, particularly in Middle Eastern countries [9]. A 2016 US study showed that after 12 months of LNG‐IUD use, women with HMB were less likely to report amenorrhoea [28]. However, the uptake of LNG‐IUDs is impacted by amenorrhoeic concerns, rejection by caregivers/patients and limited access [26]. The Madinah protocol avoids the extended use of combined hormonal therapies, often causing amenorrhoea and allows W&Gs to have their monthly periods and uses rFVIIa to control HMB. However, rFVIIa is an expensive treatment option, short‐acting and requires hospital administration. This exemplifies the need to educate W&Gs about treatment options and personalise management, considering the benefits, side effects, patient wishes and cost‐effectiveness [25].

In line with existing literature, the current study found no evidence of increased risk of miscarriage or APH. There were no cases of placental dysfunction, pre‐eclampsia or SGA. All three cases of miscarriage led to excessive bleeding despite haemostatic prophylaxis in 2/3 with known GT diagnosis. Access to an MDT in a specialised centre and careful planning of appropriate haemostatic cover may prevent excessive bleeding during miscarriage [29].

A review by Siddiq et al. demonstrated an overall 58% PPH rate in pregnancies of women with GT despite the administration of a haemostatic cover [20], correlating with the 63% rate in this cohort, with most requiring further treatment (primarily platelet transfusion). Indeed, 6/8 (75%) who received prophylactic haemostatic cover still suffered from PPH, highlighting the important need for a detailed MDT assessment of the severity of bleeding phenotype, presence of alloantibodies causing platelet refractoriness and obstetric risk factors prior to labour and delivery to plan adequate obstetric and haemostatic measures to minimise the risk of PPH. According to a guideline by the Royal College of Obstetricians and Gynaecologists, rFVIIa should be administered prophylactically at delivery, and TXA should be given from the beginning of established labour and continued through the postpartum period [30]. The efficacy of rFVIIa alone or in combination with platelet transfusion and TXA has been previously confirmed in treating PPH [31, 32, 33].

Based on the common occurrence of PPH in this population, the management plan by an MDT should include selection of the place of delivery, availability of clinicians with expertise in bleeding disorders, and ensured provision of haemostatic and blood products. One of the nine pregnancies in which the GT diagnosis was known did not receive haemostatic cover during labour and delivery due to poor antenatal planning, causing massive haemorrhage. Delivery should be planned in a specialised tertiary centre with blood bank facilities, ensuring the 24‐h availability of HLA‐matched platelets, rFVIIa and the presence of expert clinicians in the management of GT. Planned delivery at 39 weeks could be considered to facilitate this and negate unpredictable presentations in labour [29, 34].

GT diagnosis was unknown prior to seven pregnancies in three women. Of these, no PPH occurred in 4/7 pregnancies, which could be indicative of the mild bleeding phenotype in these women [20]. It is well established that the severity of bleeding in GT is unpredictable and may vary significantly between patients due to phenotypic variability, with some reaching adulthood without any severe bleeding [6, 20]. Also, PPH is multifactorial, with the commonest cause being uterine atony. However, women with bleeding disorders are at higher risk of PPH (primary and secondary) due to underlying coagulopathy.

Given that often, the first bleeding symptoms of GT appear in childhood, screening for the pathogenic variants of the *ITGA2B* and *ITGB3* genes is essential for genetic counselling and identification of those with a severe bleeding tendency that could potentially reduce the risk of PPH through appropriate preventative care [29]. Regular gynaecological follow‐up for young patients at risk of HMB may also improve clinical outcomes.

Uterine atony remains the most common cause for PPH [35], thus active management of the third stage of labour is imperative for all women with GT using adequate uterotonic agents and other surgical management if PPH occurs [36, 37]. The use of rFVIIa and antifibrinolytics is recommended for the prevention of PPH whilst avoiding platelet transfusion [14, 15]. This recommendation is also applicable to patients prior to child‐bearing age, for whom the use of antifibrinolytics, hormonal therapy and/or rFVIIa for control of menstrual bleeding is recommended to avoid platelet transfusion and risks of platelet alloimmunisation [9].

The mode of delivery should be determined by obstetric indications, considering the lack of consensus in literature [9, 34]. Because GT is an autosomal recessive inheritance disorder, provision of screening in consanguineous families/communities to identify at‐risk foetuses and counselling regarding foetal/neonatal bleeding risk, should form part of the overall management plan [3]. In this study, 25% of the neonates were later diagnosed with GT, reflecting a high rate of consanguinity in this cohort.

NAIT was reported in 31% of neonates in our study. Bleeding complications during/after birth are a recognised problem in foetuses/neonates with NAIT due to maternal alloimmunisation to human leukocyte antigen (HLA) or specifically to GP IIb/IIIa [38]. This highlights the importance of identifying HLA or GP IIb/IIIa antibodies during pregnancy (reported in ≤ 70% of patients with GT) [3, 15] to establish appropriate foetal monitoring, plans for delivery (e.g., considering planned CS if there is a high risk of bleeding), and neonatal care [34].

Registry data have become an important source of information for healthcare professionals, providing many benefits, including insights into rare diseases such as GT as well as snapshots of medical practice [39]. However, it is important to acknowledge the limitations of registry data, in particular, incomplete data capturing, lack of standardisation across registries, and small sample sizes that may impact the robustness of the findings and any conclusions made [39]. These should be addressed in future studies.

## 5 Conclusion

Despite the limitations of registry data, registries for rare disorders remain a powerful tool for healthcare professionals to improve patient care and advance medical knowledge. The current data from the ISTH REDCap registry on obstetrics and gynaecological outcomes of women with IPFD, highlight the importance of access for W&Gs with GT to an MDT and appropriate treatment options, to accommodate the range of symptoms and phenotypic severity in these W&Gs. Although HMB is very common in W&Gs with GT, it can be effectively managed with an individualised plan for a combination therapy, with consideration of cultural background, education and cost‐effectiveness. Likewise, advanced planning for an appropriate level of haemostatic cover for labour and delivery can reduce the risk and magnitude of PPH. The potential risks of alloimmunisation associated with platelet transfusions might be minimised by using rFVIIa and antifibrinolytics more efficiently. However, more research is needed to further evaluate this approach.

## Author Contributions

Rezan Abdul‐Kadir, Deborah Obeng‐Tuudah and Ahmad Tarawah designed the study. Deborah Obeng‐Tuudah, Melike Ozkan and Ahmad Tarawah performed data collection. Deborah Obeng‐Tuudah, Ahmad Tarawah and Rezan Abdul‐Kadir analysed and interpreted the data. All authors participated in the writing of the manuscript and approved the final version for submission.

## Ethics Statement

Ethic committee approvals were obtained in both institutes participating in the registry.

## Consent

The authors have nothing to report.

## Conflicts of Interest

Rezan Abdul‐Kadir, Deborah Obeng‐Tuudah, and Melike Ozkan state that they have no conflicts of interest. Ahmad Tarawah: Honorarium from Novo Nordisk for lecturing and advisory board participation.

## Supporting information



Supporting Information

## Data Availability

The survey underlying the ISTH REDCap registry for obstetric and gynaecological outcomes of women with platelet function disorders can be accessed here: https://redcap.isth.org/surveys/?s = HTHE4TAMTM.

## References

[1] D. Phillips and P. Agin , “Platelet Membrane Defects in Glanzmann's Thrombasthenia: Evidence for Decreased Amounts of Two Major Glycoproteins,” Journal of Clinical Investigation 60, no. 3 (1977): 535–545. 70433 10.1172/JCI108805 PMC372398

[2] E. I. Peerschke , M. B. Zucker , R. A. Grant , J. J. Egan , and M. M. Johnson , “Correlation Between Fibrinogen Binding to Human Platelets and Platelet Aggregability,” Blood 55, no. 5 (1980): 841–847. 6767512

[3] J. P. Botero , K. Lee , B. R. Branchford , et al., “Glanzmann Thrombasthenia: Genetic Basis and Clinical Correlates,” Haematologica 105, no. 4 (2020): 888–894. 32139434 10.3324/haematol.2018.214239 PMC7109743

[4] A. T. Nurden , M. Fiore , P. Nurden , and X. Pillois , “Glanzmann Thrombasthenia: A Review of ITGA2B and ITGB3 Defects With Emphasis on Variants, Phenotypic Variability, and Mouse Models,” Blood, Journal of the American Society of Hematology 118, no. 23 (2011): 5996–6005. 10.1182/blood-2011-07-365635 21917754

[5] R. A. Tarawah and A. M. Tarawah , “Pregnancy and Delivery Outcome Among Ladies With Glanzmann Thrombasthenia: A Report From Glanzmann Thrombasthenia Registry of Al‐Madinah, Saudi Arabia,” Blood 142 (2023): 3968.

[6] J. N. George , J. P. Caen , and A. T. Nurden , “Glanzmann's Thrombasthenia: The Spectrum of Clinical Disease,” Blood 75, no. 7 (1990): 1383–1395. 2180491

[7] National Institute for Health and Care Excellence (NICE) Guideline . Heavy Menstrual Bleeding: Assessment and Management. Published: 14 March 2018 Last updated: 24 May 2021. Accessed October 30, 2023, https://www.nice.org.uk/guidance/ng88.

[8] B. S. Apgar , A. H. Kaufman , U. George‐Nwogu , and A. Kittendorf , “Treatment of Menorrhagia,” American Family Physician 75, no. 12 (2007): 1813–1819. 17619523

[9] A. Tarawah , T. Owaidah , N. Al‐Mulla , et al., “Management of Glanzmann's Thrombasthenia; Guidelines Based on an Expert Panel Consensus From Gulf Cooperation Council Countries,” Journal of Applied Hematology 10, no. 1 (2019): 1–9.

[10] F. Rodeghiero , A. Tosetto , T. Abshire , et al., “ISTH/SSC Bleeding Assessment Tool: A Standardized Questionnaire and a Proposal for a New Bleeding Score for Inherited Bleeding Disorders,” Journal of Thrombosis and Haemostasis 8, no. 9 (2010): 2063–2065. 20626619 10.1111/j.1538-7836.2010.03975.x

[11] M.‐C. Poon , R. d'Oiron , R. B. Zotz , N. Bindslev , M. N. D. Di Minno , and G. Di Minno , “The International, Prospective Glanzmann Thrombasthenia Registry: Treatment and Outcomes in Surgical Intervention,” Haematologica 100, no. 8 (2015): 1038. 26001792 10.3324/haematol.2014.121384 PMC5004419

[12] G. Di Minno , R. B. Zotz , R. d'Oiron , N. Bindslev , M. N. D. Di Minno , and M.‐C. Poon , “The International, Prospective Glanzmann Thrombasthenia Registry: Treatment Modalities and Outcomes of Non‐Surgical Bleeding Episodes in Patients With Glanzmann Thrombasthenia,” Haematologica 100, no. 8 (2015): 1031. 26001793 10.3324/haematol.2014.121475 PMC5004418

[13] M. C. Poon , R. d'Oiron , M. Von Depka , et al., “Prophylactic and Therapeutic Recombinant Factor VIIa Administration to Patients With Glanzmann's Thrombasthenia: Results of an International Survey,” Journal of Thrombosis and Haemostasis 2, no. 7 (2004): 1096–1103. 15219192 10.1111/j.1538-7836.2004.00767.x

[14] J. Alamelu and R. Liesner , “Modern Management of Severe Platelet Function Disorders,” British Journal of Haematology 149, no. 6 (2010): 813–823. 20456364 10.1111/j.1365-2141.2010.08191.x

[15] P. H. Bolton‐Maggs , E. A. Chalmers , P. W. Collins , et al., “A Review of Inherited Platelet Disorders With Guidelines for Their Management on Behalf of the UKHCDO,” British Journal of Haematology 135, no. 5 (2006): 603–633. 17107346 10.1111/j.1365-2141.2006.06343.x

[16] M.‐C. Poon , G. Di Minno , R. d'Oiron , and R. Zotz , “New Insights Into the Treatment of Glanzmann Thrombasthenia,” Transfusion Medicine Reviews 30, no. 2 (2016): 92–99. 26968829 10.1016/j.tmrv.2016.01.001

[17] M. C. Poon , “The Use of Recombinant Activated Factor VII in Patients With Glanzmann's Thrombasthenia,” Thrombosis and Haemostasis 121, no. 3 (2021): 332–340. 33124022 10.1055/s-0040-1718373 PMC7895543

[18] M.‐C. Poon , R. d'Oiron , S. Baby , R. B. Zotz , and G. J. H. Di Minno , “The Glanzmann Thrombasthenia Registry: Safety of Platelet Therapy in Patients With Glanzmann Thrombasthenia and Changes in Alloimmunization Status,” Haematologica 108, no. 10 (2023): 2855–2858. 36924249 10.3324/haematol.2022.281973 PMC10542831

[19] S. A. Faiz and M. Shakeel , “Glanzmann's Thrombasthenia in Pregnancy; a Case and Review of the Literature,” Journal of the Society of Obstetricians and Gynaecologists of Pakistan 8, no. 2 (2018): 137–139.

[20] S. Siddiq , A. Clark , and A. Mumford , “A Systematic Review of the Management and Outcomes of Pregnancy in Glanzmann Thrombasthenia,” Haemophilia 17, no. 5 (2011): e858–e869. 21457404 10.1111/j.1365-2516.2011.02516.x

[21] International Society on Thrombosis and Haemostasis (ISTH) REDCap Registry Survey. OB/GYN Outcomes of Women With Platelet Function Disorders . Accessed August 31, 2023, https://redcap.isth.org/surveys/?s=HTHE4TAMTM.

[22] R. Kadir , M. Edlund , and S. Von Mackensen , “The Impact of Menstrual Disorders on Quality of Life in Women With Inherited Bleeding Disorders,” Haemophilia 16, no. 5 (2010): 832–839. 20584085 10.1111/j.1365-2516.2010.02269.x

[23] N. T. Connell , V. H. Flood , R. Brignardello‐Petersen , et al., “ASH ISTH NHF WFH 2021 Guidelines on the Management of von Willebrand Disease,” Blood Advances 5, no. 1 (2021): 301–325. 33570647 10.1182/bloodadvances.2020003264 PMC7805326

[24] N. Curry , L. Bowles , T. J. Clark , et al., “Gynaecological Management of Women With Inherited Bleeding Disorders. A UK Haemophilia Centres Doctors' Organisation Guideline,” Haemophilia 28, no. 6 (2022): 917–937. 35976756 10.1111/hae.14643

[25] E. P. Mauser‐Bunschoten , R. A. Kadir , E. T. Laan , et al., “Managing Women‐Specific Bleeding in Inherited Bleeding Disorders: A Multidisciplinary Approach,” Haemophilia 27, no. 3 (2021): 463–469. 33314402 10.1111/hae.14221 PMC8246991

[26] M. A. Parks , N. Zwayne , and M. Temkit , “Bleeding Patterns Among Adolescents Using the Levonorgestrel Intrauterine Device: A Single Institution Review,” Journal of Pediatric and Adolescent Gynecology 33, no. 5 (2020): 555–558. 32376363 10.1016/j.jpag.2020.04.006

[27] K. van Galen , M. Lavin , N. Skouw‐Rasmussen , et al., “European Principles of Care for Women and Girls With Inherited Bleeding Disorders,” Haemophilia 27, no. 5 (2021): 837–847. 34343384 10.1111/hae.14379

[28] M. Mejia , C. McNicholas , T. Madden , and J. F. Peipert , “Association of Baseline Bleeding Pattern on Amenorrhea With levonorgestrel Intrauterine System Use,” Contraception 94, no. 5 (2016): 556–560. 27364099 10.1016/j.contraception.2016.06.013 PMC5077249

[29] M. Fiore , L. Sentilhes , and R. d'Oiron , “How I Manage Pregnancy in Women With Glanzmann Thrombasthenia. *Blood* ,” Journal of the American Society of Hematology 139, no. 17 (2022): 2632–2641. 10.1182/blood.2021011595 35286390

[30] S. Pavord , R. Rayment , B. Madan , T. Cumming , W. Lester , and E. Chalmers , “Management of Inherited Bleeding Disorders in Pregnancy: Green‐Top Guideline no. 71 (Joint With UKHCDO),” BJOG 124, no. 8 (2017): e193–e263. 28447403 10.1111/1471-0528.14592

[31] J. Ahonen and R. Jokela , “Recombinant Factor VIIa for Life‐Threatening Post‐Partum Haemorrhage,” British Journal of Anaesthesia 94, no. 5 (2005): 592–595. 15708871 10.1093/bja/aei094

[32] F. Moscardó , F. Pérez , J. D. L. Rubia , et al., “Successful Treatment of Severe Intra‐Abdominal Bleeding Associated With Disseminated Intravascular Coagulation Using Recombinant Activated Factor VII,” British Journal of Haematology 114, no. 1 (2001): 174–176. 11472364 10.1046/j.1365-2141.2001.02878.x

[33] F. W. Bouwmeester , A. R. Jonkhoff , R. H. Verheijen , and H. P. van Geijn , “Successful Treatment of Life‐Threatening Postpartum Hemorrhage With Recombinant Activated Factor VII,” Obstetrics and Gynecology 101, no. 6 (2003): 1174–1176. 12798521 10.1016/s0029-7844(03)00350-8

[34] G. Sarpietro , R. L. Grimaldi , R. Brescia , M. G. Matarazzo , and A. Cianci , “Glanzmann's Thrombasthenia During Pregnancy: Case Report and Literature Review,” SN Comprehensive Clinical Medicine 5, no. 1 (2023): 1–8.36407770

[35] Y. Oyelese and C. V. Ananth , “Postpartum Hemorrhage: Epidemiology, Risk Factors, and Causes,” Clinics in Obstetrics and Gynaecology 53, no. 1 (2010): 147–156. 10.1097/GRF.0b013e3181cc406d 20142652

[36] L. Sentilhes , N. Winer , E. Azria , et al., “Tranexamic Acid for the Prevention of Blood Loss After Vaginal Delivery,” New England Journal of Medicine 379, no. 8 (2018): 731–742. 30134136 10.1056/NEJMoa1800942

[37] L. Sentilhes , M. V. Sénat , M. Le Lous , et al., “Tranexamic Acid for the Prevention of Blood Loss After Cesarean Delivery,” New England Journal of Medicine 384, no. 17 (2021): 1623–1634. 33913639 10.1056/NEJMoa2028788

[38] N. Leticee , C. Kaplan , and D. Lemery , “Pregnancy in Mother With Glanzmann's Thrombasthenia and Isoantibody Against GPIIb‐IIIa: Is There a Foetal Risk?” European Journal of Obstetrics, Gynecology, and Reproductive Biology 121, no. 2 (2005): 139–142. 16054952 10.1016/j.ejogrb.2005.02.011

[39] E. C. Nelson , M. Dixon‐Woods , P. B. Batalden , et al., “Patient Focused Registries Can Improve Health, Care, and Science,” BMJ 354 (2016): i3319. 27370543 10.1136/bmj.i3319 PMC5367618

