# Supplementary material for: Obstetric and Gynaecological Challenges and Outcomes in Women and Girls With Glanzmann's Thrombasthenia
Source: Haemophilia. 2025 Mar 23;31(3):509–18. doi: 10.1111/hae.70030 (PMC12175114; doi:10.1111/hae.70030)
Supplement: Supplementary file 1 — Supporting Information [file HAE-31-509-s001.docx]

Supplementary data

Supplementary Table S1: Number of women and girls with Glanzmann’s thrombasthenia treated effectively for acute HMB (n=31).

| Treatment agents | Number of patients, n/n (%) |
| --- | --- |
| Hormonal therapy, n (%) | 30 (96.8) |
| Platelets, n (%)  No. of units, median (range) | 18 (58.1)  8 (1–29) |
| Antifibrinolytics, n (%) | 17 (54.8) |
| Iron treatment (oral or intravenous), n (%) | 17 (54.8) |
| pRBCs, n (%)  No. of units, median (range) | 16 (51.6)  2.5 (1–19) |
| Combined oral contraceptive pill, n (%) | 9 (29) |
| rFVIIa, n (%) | 4 (12.9) |
| Surgery^†^, n (%) | 1(3) |

HMB, heavy menstrual bleeding; n, number of patients; pRBCs, packed red blood cells; rFVIIa, recombinant factor VIIa; TXA, tranexamic acid.
^†^Surgical intervention consisted of hysteroscopy with dilation and curettage.

## Supplementary Table S2: Outcomes in neonates

| **Neonatal characteristics and outcomes** | |
| --- | --- |
| **Total, N**  **Male, n (%)**  **Female, n (%)** | 16  8 (50%)  8 (50%) |
| **Diagnosed with GT, n (%)** | 4 (25%) |
| **Admitted to NICU**  **Reason:**  **Mother CS****^†^**  **Observation**  **Mother in HDU**  **Subdural haematoma**  **Oozing**  **Intracranial haemorrhage^‡^** | 9/16 (56%)  3  2  1  1  1  1 |
| **Days of neonatal hospitalisation, median (range)** | 7 (9–30) |
| **Neonatal alloimmune thrombocytopenia, n (%)** | 5 (31%) |

CS, caesarean section; GT, Glanzmann’s thrombasthenia; HDU, high dependency unit;
n, number; N, total number; NICU, neonatal intensive care unit.

**^†^**of which one patient also had low platelets

^‡^treated with platelet transfusion, packed red blood cells, and intravenous immunoglobulin
